# What Do We Know about Nociplastic Pain?

**DOI:** 10.3390/healthcare11121794

**Published:** 2023-06-17

**Authors:** Kacper Bułdyś, Tomasz Górnicki, Dariusz Kałka, Ewa Szuster, Małgorzata Biernikiewicz, Leszek Markuszewski, Małgorzata Sobieszczańska

**Affiliations:** 1 Faculty of Medical Sciences and Health Sciences, Kazimierz Pulaski University of Technology and Humanities in Radom, 26-600 Radom, Poland; 2 Faculty of Medicine, Wroclaw Medical University, 50-367 Wroclaw, Poland; 3 Faculty of Physiotherapy, Wroclaw University of Health and Sport Sciences, 51-612 Wroclaw, Poland; 4 Men’s Health Centre in Wrocław, 53-151 Wroclaw, Poland; 5 Cardiosexology Students Club, Wroclaw Medical University, 50-368 Wroclaw, Poland; 6 Clinical Department of Geriatrics, Wroclaw Medical University, 50-369 Wroclaw, Poland

**Keywords:** nociplastic pain, chronic pain, management, diagnosis

## Abstract

Nociplastic pain is a recently distinguished type of pain, distinct from neuropathic and nociceptive pain, and is well described in the literature. It is often mistaken for central sensitization. Pathophysiology has not been clearly established with regard to alteration of the concentration of spinal fluid elements, the structure of the white and gray matter of the brain, and psychological aspects. Many different diagnostic tools, i.e., the painDETECT and Douleur Neuropathique 4 questionnaires, have been developed to diagnose neuropathic pain, but they can also be applied for nociplastic pain; however, more standardized instruments are still needed in order to assess its occurrence and clinical presentation. Numerous studies have shown that nociplastic pain is present in many different diseases such as fibromyalgia, complex regional pain syndrome type 1, and irritable bowel syndrome. Current pharmacological and nonpharmacological treatments for nociceptive and neuropathic pain are not entirely suitable for treating nociplastic pain. There is an ongoing effort to establish the most efficient way to manage it. The significance of this field has led to several clinical trials being carried out in a short time. The aim of this narrative review was to discuss the currently available evidence on pathophysiology, associated diseases, treatment possibilities, and clinical trials. It is important that physicians widely discuss and acknowledge this relatively new concept in order to provide optimized pain control for patients.

## 1. Introduction

The International Association for the Study of Pain (IASP) defines pain as “an unpleasant sensory and emotional experience associated with or resembling that associated with, actual or potential tissue damage” [1]. The term nociplastic pain (NcplP) was first mentioned and introduced in 2016 as a new concept, different from the well-described nociceptive and neuropathic pain [2]. Currently, NcplP is defined as “pain that arises from altered nociception despite no clear evidence of actual or threatened tissue damage that causes peripheral nociceptors activation or evidence of disease or lesion of the somatosensory system causing the pain” [1]. Due to the novelty of this concept, it is difficult to define this phenomenon, creating a diagnostic problem for physicians [3,4,5,6]. Researchers are constantly debating the nature of NcplP but have not reached any agreement yet [2,7,8,9]. This pain is often considered to be a part or counterpart of central sensitization; however, some authors present arguments against such an idea [3,7,10]. There is a vast group of diseases in which NcplP plays an important role, with fibromyalgia being the most commonly mentioned [11]. With the introduction of the ICD-11, there is an incoherence in NcplP’s presence in this classification [5,12]. Keeping in mind that the term “nociplastic pain” is fairly new, we used a simple search strategy to find proper articles. We decided to search for the phrase “nociplastic pain” through recognized databases. To be included, the review or at least the abstract had to be written in English and published after the introduction of the term “nociplastic pain”. Furthermore, there is no record of “nociplastic pain” in MeSH terms in the PubMed database. Taking into account the occurrence of an inconsistent approach to NcplP, we conducted a narrative review with the aim of summarizing the current state of knowledge on the pathophysiology of NcplP, comorbidities, diagnostic tools, and possible therapies. We also searched for ongoing clinical trials covering this condition. The purpose of this work was to review scientific evidence in order to better understand the concept of NcplP. To the best of our knowledge, this is the first article to discuss this topic.

**Figure 1:**
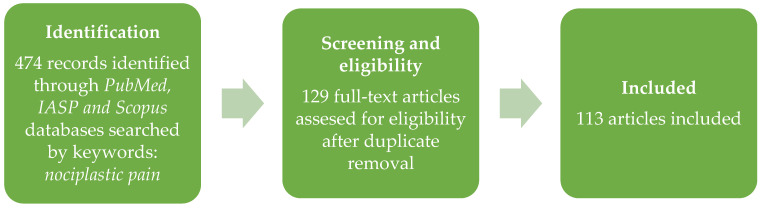
Flow diagram.

The search was conducted in the PubMed, IASP, and Scopus databases. Of the 474 identified records, 113 articles were included. A simplified flow diagram of study selection is depicted in Figure 1.

## 2. Pathophysiology

NcplP is a type of pain in which there is no tissue damage that can activate nociceptors or any other evidence of somatosensory disease [13]. It appears in chronic pain conditions such as headaches, fibromyalgia, and low back pain (LBP) [14].

Researchers have proposed several mechanisms underlying NcplP [15]. Taking into account the strong but preliminary evidence on currently known mechanisms, they can be divided into three categories. The first category includes supraspinal mechanisms such as hyperresponsiveness to pain stimuli, hyperactivity, and connectivity between regions of the brain responsible for pain perception, for example, the medial prefrontal cortex and rostral (mPFC), the anterior cingulate cortex (ACC), and the thalamus and secondary somatosensory cortices [15,16]. Additionally, decreased activity and connectivity of brain areas responsible for pain inhibition, that is, the connection between mPFC and ACC and insula, are believed to be present in NcplP. Other supraspinal mechanisms of NcplP include an increased concentration of substance P and glutamine levels in cerebrospinal fluid and inhibition of GABAergic transmission [15]. There is also evidence of a fluctuation in the size and shape of gray and white matter in areas related to pain. The second category includes spinal mechanisms [17]. This group involves regional clustering and convergence of signals from different pain loci, spinal cord reorganization, amplified spinal reflex transmission, decreased spinal inhibition, winding up and temporal summation, and immune system activation among other glial cells (Table 1) [15,17,18,19,20,21,22]. The third category includes peripheral mechanisms that are related to the proliferation of sodium channels and sympatho-afferent coupling [15]. In clinical practice, pain signaling involves main compartments that can contribute to the development of pain in all three sites: supraspinal, spinal, and peripheral [23,24]. As an example, patients with Parkinson’s disease can experience pain related to Parkinson’s disease and pain unrelated to Parkinson’s disease [25]. However, in Parkinson’s disease, NcplP seems to be triggered by a dopamine agonist withdrawal syndrome or dopaminergic dysregulation syndrome linking the occurrence of NcplP with dopaminergic transmission [26].

**Table 1:** Currently known pathophysiological mechanisms of nociplastic pain.

Category	Specific Mechanisms	References
Supraspinal mechanisms	Hyperresponsiveness to pain stimuli Hyperactivity and connectivity between mPFC, ACC, thalamus, secondary somatosensory cortices Decreased activity and connectivity between mPFC, ACC, and insula Increased concentration of substance P and glutamine level in cerebrospinal fluid Inhibition of GABAergic transmission Fluctuation in the size and shape of gray and white matter of the brain Higher level of primary and secondary emotions Parkinson’s-disease-related pain Dopamine agonist withdrawal syndrome Dopaminergic dysregulation syndrome Brain plasticity	[15,18,20,21,22,27,28,29]
Spinal mechanisms	Regional clustering and convergence of signals from different pain loci Spinal cord reorganization Amplified spinal reflex transmission Diminished spinal inhibition Wind-up and temporal summation Immune system activation, e.g., glial cells	[15,18,20,21,22,27]
Peripheral mechanisms	Proliferation of sodium channels Sympatho-afferent coupling	[15]

Considering the origin of NcplP, the most probable cause is based on a biopsychosocial model that indicates a variety of backgrounds that trigger and predispose to NcplP, including a history of abuse, environmental exposure, and genetic and epigenetic alterations of the genome [15,30]. A study conducted on the mouse model of NcplP showed that in female mice, it was easier to induce NcplP than in male mice. Additionally, differences in how pain is mediated were observed between male and female experimental pain models. Male mice manifested greater mechanical hypersensitivity in comparison to female mice and differences in response to treatment. Microglia in the spinal cord play a role in the mechanism of chronic mechanical hypersensitivity in the male nociplastic pain model. However, in the female model, silencing afferent activity at the postinjury site reduced hypersensitivity outside of the injured area. Altogether, these data provide evidence that inducing and regulating pathways may be sex-related [28,29]. Other studies indicate that brain plasticity is responsible for NcplP induction. The occurrence and intensity of NcplP can also be correlated with a higher level of primary emotions, for example, anger, and secondary emotions, which are emotional reactions to other emotions [28]. A study conducted on patients with LBP revealed the variability of the organization of the primary motor cortex, concluding that this difference is the origin of NcplP in this condition [31,32]. The level of C-reactive protein can also be associated with NcplP, as shown in a study conducted on patients with fibromyalgia in which a correlation between the C-reactive protein level and severity of pain was reported [33].

## 3. Diagnosis

When diagnosing NcplP, it is crucial to be able to objectively confirm its occurrence. In 2021, the IASP announced the set of clinical criteria and a grading system for NcplP. Pain, in order to be classified as NcplP, has to have specific features which must be identified regarding its characteristics, hypersensitivity, and the presence of comorbidities. Awareness of NcplP characteristics facilitates the clinical reasoning process [11]. In line with the IASP statement, the current literature provides evidence that the diagnosis of NcplP is a highly complex process, and that the gold standard has not been developed yet [34,35]. A mnemonic RATE (recognize, assess, treat, and evaluate) strategy has been proposed for the identification of patients with chronic pain, and is also useful for patients suspected of having NcplP [36]. First, a comprehensive physical examination and past medical history should be performed, as some details from a patient’s history can suggest NcplP, such as a significantly increased use of healthcare services [15,37]. Furthermore, patients suffering from NcplP are more likely to report dull, fluctuating, widespread pain [37].

Various outcome measures, listed in Table 2, can be used in preliminary examination or screening for the occurrence of NcplP in differential diagnosis and to tailor treatment [15,31,38]. Although quantitative sensory testing (QST) is an umbrella term that includes various tests, most of the time, diagnosing NcplP requires a very specific approach. The QST is used as a tool in research rather than in clinical practice [15,39]. It focuses on factors such as pain pressure thresholds, conditioned pain modulation, and temporal summation. It is important to emphasize the offset analgesia phenomenon when QST is performed [11,40]. Nonetheless, qualitative sensory tests have also been mentioned as a method of assessing NcplP in orofacial pain conditions [41]. Some inconsistency can be noted when comparing different diagnostic criteria for pain assessment in fibromyalgia, i.e., the 1990 American College of Rheumatology (ACR) criteria [32,42], the 2011 ACR Fibromyalgia Survey criteria [15,20,38,43,44,45], and the 2016 ACR criteria [33,37,46,47].

Assessment of sleep quality was also conducted using different tools, i.e., the Pittsburgh Sleep Quality Index, the Leeds Sleep Evaluation Questionnaire, the Insomnia Severity Index, the Medical Outcomes Study Sleep Scale, and wrist actigraphy [15,26,48]. Lastly, although various tools are reported in the literature, clinical reasoning and physician experience are a key success factors in the diagnosis of NcplP, as many patients can present a mixture of pain phenotypes, e.g., nociceptive and nociplastic pain, at the same time [15].

The diagnostic process in NcplP is mainly focused on excluding other conditions [49]; however, this approach can often leads to a misdiagnosis of NcplP [15]. In addition, to confirm the occurrence of NcplP, instruments to exclude nociceptive and neuropathic pain are used as well [13,15,26,46,50]. In the case of diseases associated with the confirmed presence of NcplP, there are specific diagnostic protocols or criteria to evaluate their symptoms [11,13,25,35,38]. For example, the Central Sensitization Inventory (CSI) can not only give an indication of central sensitization but also of comorbidities, according to the IASP criteria [51]. On top of this, some reports postulate that some scales and questionnaires are of low accuracy [29,41,52].

To date, no specific laboratory markers have been identified to provide a clear distinction between NcplP and other types of pain [35,53]. One report suggested that inflammation may contribute to the occurrence of NcplP. Lower serum tryptophan and tryptophan-kynurenine metabolic pathways are also linked to neuroinflammation [22]. A lower concentration of serum brain-derived neurotrophic factor was reported in patients with fibromyalgia, but a difference between NcplP and nociceptive pain was not significant [54].

There have been attempts to use diagnostic imaging such as functional neuroimaging, activation pattern and brain mapping with the use of positron emission tomography, magnetic resonance imaging, and electromyography [11,15,19,20,31,32,46,55]. According to a study using magnetic resonance imaging, treatment was shown to reverse the brain changes caused by LBP [56].

Evaluating the sensory profile of a patient might be helpful in assessing symptoms [35,41]. The existence of a distinguishing characteristic for primary musculoskeletal pain that is predominantly nociplastic is theorized. The suggested criteria are comprehensive and allow for a step-by-step analysis of a patient’s condition [57]. In addition to that, in 2021, the IASP released clinical criteria for NcplP [10]. Other recently proposed methods of assessing NcplP are the Skorupska Protocol (SP) [4,58] and Nociplastic-based Fibromyalgia Features (NFF) [59]. The NFF criteria are focused on NcplP attributes and were reported to be helpful in the diagnosis of fibromyalgia [60]. Taking SP into account, it is a stress test during which an atypical vasomotor reaction may be observed with the use of an infrared thermal camera [58].

It is important to mention that the NcplP is a suitable phenotype not only for use in adult patients, but there is also evidence that this term is suitable for describing pain in children and adolescents [61]. Children and adolescents suffering from NcplP more often present symptoms of panic disorder and social phobia, and have worse quality of sleep in comparison to patients with other types of pain [61]. It is also stated that in rheumatic diseases, NcplP in children has a stronger genetic component than in older individuals [62].

**Table 2:** Most common outcome measures used to assess pain in patients based on analyzed in the literature.

No.	Type	Full Tool Name	Abbreviation	Reference
		Overall health assessment		
1	Questionnaire	Fibromyalgia Impact Questionnaire	FIQ	[63]
2	Questionnaire	McGill Pain Questionnaire	MPQ	[25]
3	Questionnaire	Revised Fibromyalgia Impact Questionnaire	FIQR	[54]
4	Questionnaire	Pittsburgh Sleep Quality Index	PSQI	[64]
5	Questionnaire	General Health Questionnaire	GHQ	[8]
6	Questionnaire	Patient Health Questionnaire	PHQ	[65]
7	Questionnaire	Short-Form Health Survey	SF	[34]
8	Scale	Patient-Reported Outcomes Measurement Information System	PROMIS	[45]
		Psychosocial assessment		
1	Questionnaire	Hospital Anxiety and Depression Scale	HADS	[66]
2	Questionnaire	Beck Depression Inventory	BDI	[34]
3	Questionnaire	State and Trait Anxiety Inventory	STALDY	[32]
4	Scale	Pain Catastrophizing Scale	PCS	[67]
5	Scale	Tampa Scale for Kinesiophobia	TSK	[68]
6	Scale	Hamilton Depression Rating Scale	HAM-D	[34]
7	Scale	King’s Parkinson’s Disease Pain Scale	KPPS	[25]
8	Scale	Pain Anxiety Symptoms Scale	PASS	[35]
		Pain assessment		
1	Questionnaire	Pain Self-Efficacy Questionnaire	PSEQ	[66]
2	Questionnaire	Örebro Musculoskeletal Pain Screening Questionnaire	ÖMPQ	[31]
3	Questionnaire	Douleur Neuropathique 4	DN4	[13]
4	Questionnaire	painDETECT	PD-Q	[13]
5	Questionnaire	McGill Pain Questionnaire	MPQ	[25]
6	Scale	Leeds Assessment of Neuropathic Symptoms and Signs	LANSS	[69]
7	Scale	Numerical Rating Scale	NRS	[68]
8	Scale	Visual Analog Scale	VAS	[60]
9	Scale	Central Sensitization Inventory	CSI	[51]
10	Test	Quantitative Sensory Testing	QST	[11]
11	Test	FibroDetect Test	N/A	[50]
12	Tool	Brief Pain Inventory	BPI	[25]
13	Tool	Neuropathic Pain Special Interest Group algorithm	NeuPSIG	[70]
14	Tool	Fibromyalgia Rapid Screening Tool	FiRST	[50]
15	Tool	STarT Back Screening Tool	SBT	[55]
16	Tool	Nociplastic-based Fibromyalgia Features	NFF	[59]

## 4. Diseases Associated with NcplP

NcplP has been reported to be part of symptomatology in many different diseases with various pathophysiology and causes. In order to provide a clear presentation of this topic, we decided to use a modified classification developed by Fitzcharles et al. [15]. Fibromyalgia is the most frequently reported health condition associated with NcplP, often used as a synonym. Researchers claim that fibromyalgia often occurs with other comorbidities [13,15,37,45,46,59,71]. NcplP described in fibromyalgia is reported to be caused by a central sensitization mechanism [15]. However, some studies found neuropathic pain in fibromyalgia, using the term “fibromyalgianess” for the overall health condition of those patients [6,13,20,54,71]. This term was introduced to establish a patient-adjusted scale which takes into account differences between individuals and links the clinical picture with various diagnostic scales [72].

Regarding the musculoskeletal group, currently, there is no clear consensus on whether musculoskeletal pain and LBP have a component of NcplP. Musculoskeletal pain is a field of research due to its potentially nociplastic nature [13,29]. In LBP [13,14,15,19,22,29,37,53,55,69,73,74] and primary musculoskeletal pain [15,59,60], the nociplastic component of pain is often studied as well. There is a debate about whether complex regional pain syndrome (CRPS) has features of NcplP [18]. Some researchers hypothesize that NcplP in musculoskeletal pain is due to age-related changes in the spine; however, this claim does not seem to be strongly supported by evidence [15,75]. Interestingly, type 1 CRPS, is caused by NcplP and some articles even define it as “nociplastic pain syndrome” [15,76]. In some other reports, the type of CRPS was omitted, which makes conclusions difficult to draw [19,53,77].

Pain is a dominant symptom of rheumatic diseases. Although is it mainly neuropathic and caused by mechanical injuries in the course of disease or the inflammatory process, it can also have a nociplastic component secondary to a central sensitization mechanism [78]. In patients with osteoarthritis and rheumatoid arthritis, questionnaires such as the QST and CSI can be useful to identify central sensitization; however, using them in clinical practice can be problematic due to time and cost requirements [11,46,51].

Another disease associated with NcplP is chronic visceral pain syndrome, with chronic primary visceral pain being the main symptom [15,53,59,76,79]. Chronic pelvic pain may range from the nociceptive to the nociplastic type of pain, with overlapping phenotypes [15,37,65,80]. Irritable bowel syndrome is often mentioned when talking about NcplP as well [11,15,37,69,81,82].

Chronic headaches often have features of NcplP and can be classified as chronic primary headaches, orofacial pain, tension-type headaches, and migraines [14,15,19,46,53,83]. Orofacial pain with the characteristic symptoms of NcplP can be a part of temporomandibular joint dysfunction and burning mouth syndrome [8,15,19,22,41,53,82,83,84]. Furthermore, shoulder and neck pain can develop in patients with primary myofascial pain or in breast cancer survivors. Although research suggests that NcplP is not the only component of pain in this group of patients, careful assessment of pain can help apply targeted and effective treatment [85,86].

Some articles provide data that psychosocial disorders may play a role in the development of NcplP [87,88]. Stress, disability, depression, and anxiety may cause NcplP [11,22,89,90]. However, other scientific reports do not see an increased occurrence of NcplP in patients with depression or anxiety [38]. It is worth mentioning that both fibromyalgia and depression can be rooted in neuroinflammation [91]. NcplP in cancer patients is discussed as a consequence of the nature of cancer disease or the type of treatment applied, i.e., anti-hormone therapy [27,37,85,91,92,93]. Cancer survivors are also at risk of NcplP occurrence. Nijs et al. proposed a stepwise clinical decision-making tree for NcplP [10,94]. In a post-treatment group of breast and colon cancer patients, a specific regional pain distribution was reported, which suggested the presence of NcplP [94]. The pain setting mentioned above must be more widespread than can be explained by the identifiable source of nociception [94]. It is important to exclude, if possible, the presence of metastatic disease [94]. Numerous other health conditions are mentioned in the literature as potentially associated with NcplP, including rheumatoid arthritis [13,95], osteoarthritis [96,97], gluteal syndrome [98], electrical injury [99], multiple sclerosis [43,100], cerebral palsy and spina bifida [101], Parkinson’s disease, [26] and post-COVID pain [102,103]. Some articles also mention endometriosis as a cause of NcplP [104,105]. Interestingly, in patients infected with the human T cell lymphotropic virus type 1 (HTLV-1), the virus was associated with greater intensity and characteristics of NcplP [70]. In one paper, the expression of specific genes was shown to be a possible cause and predisposition to NcplP [106].

## 5. Treatment of NcplP

Knowledge about the prevalence and characteristics of NcplP as well as its underlying mechanisms can help in tailoring treatment strategies [44], although, to date, no gold standard for treating NcplP has been developed [34,82]. The main goal of treatment is to reduce symptoms and improve quality of life [64]. Several investigators have reported that a non-pharmacological approach is most likely to be effective in the therapy of NcplP [15,36,37,45,46,55,86]. The physiotherapeutic approach is reported to be effective as an element of comprehensive treatment [86] because patients with NcplP are likely to respond better to centrally than peripherally targeted therapies [11]. Cognitive behavioral therapy (CBT) has been proven to be effective as well [36,45,55]. Emotional awareness and expression therapy (EAET) has been proven to be a helpful addition to primary therapy as it has been shown to reduce the severity of pain and other coexisting symptoms. CBT and EAET are comparable in terms of efficacy [45]. Furthermore, EAET was recommended as the treatment of choice in NcplP [19,45,107]. Acceptance and commitment therapy has also been assessed, but has not been shown to be effective in reducing pain [55]. Exercise and weight control are important to maintain patient well-being. Obesity has been reported to be a risk factor for fibromyalgia [45]. A meta-analysis that evaluated different exercise treatments for chronic pain along the continuum of NcplP found that exercise interventions can be a useful component of a tailored treatment approach [108]. For patients with NcplP, a balanced daily routine can help with pain management. Currently, data on specific diets that would help to reduce pain are insufficient [45]. Massage and acupuncture, in addition to exercise or education, have also been shown to be beneficial in the treatment of NcplP [45,55].

Pharmacological treatment is commonly used to treat pain; however, its effectiveness depends on the type of pain. Non-steroidal anti-inflammatory drugs (NSAID), paracetamol, opioids and muscle relaxants have been reported to be less effective in NcplP than in nociceptive pain or other types of pain [15,43,45]. A Cochrane review on NSAID therapy in fibromyalgia states that “NSAIDs cannot be regarded as useful for treating fibromyalgia” [109]. Another Cochrane review showed that, in the case of LBP, there are no significant differences between selective and non-selective NSAIDs [110]. In the case of opioid therapy, a weak analgesic effect has been observed only in fibromyalgia—the most common nociplastic-associated condition. The following mechanism based on disorganization of endogenous opiate receptors associated with high opioidergic tone and downregulation of MORs, the phasic release of endogenous opioids that fails to inhibit the GABA neurons and block stimulation of the antinociceptive neurons was proposed. “This phenomenon prevents the endogenous system from modulating pain in fibromyalgia” [111]. Additionally, opioid therapy may exacerbate pain in patients with fibromyalgia and other nociplastic pain-related conditions [37].

Tricyclic antidepressants (TCAs), serotonin-norepinephrine reuptake inhibitors (SNRIs), and gabapentinoids have been recognized as another potential group of treatment agents [50]. TCAs have been reported to be effective in the treatment of NcplP; however, there are some differences across studies. They were reported to be more effective in pain reduction than SNRIs but in older patients, they were associated with a higher risk of adverse effects [15,43,45,46,82]. SNRIs have been reported to have a positive effect on patients with NcplP conditions, even greater than NSAIDs, but unfortunately, clinical research has led to contradictory conclusions. However, it is important to consider a potential increase in the occurrence of adverse effects [15,26,36,43,45,46,82]. Some papers report that gabapentinoids may relieve NcplP, whereas others report the opposite results [15,43,46]. Furthermore, pregabalin is recommended by the FDA for fibromyalgia [112]. Benzodiazepines and codeine are of particular interest because they have been reported to increase the risk of prescription opioid misuse when treating NcplP [113]. Antiepileptic drugs can be considered a potential treatment [36,43,114]. It is worth mentioning that ketamine has been reported to be effective in pain reduction in CRPS 1. Unfortunately, it is limited to CRPS and fibromyalgia, so its use requires further research [76,115]. Some researchers report potential benefits of procedural types of treatment such as steroid injections for temporomandibular disorders [27], normal saline injections [100], transcranial direct current stimulation, and repetitive transcranial magnetic stimulation [87]. Although they were reported to be less effective in NcplP in comparison to other types of pain [27]; they can be administered if other interventions have been ineffective [55]. The pain relief effects of vitamin B12 may be useful as an auxiliary treatment for NcplP [47]. The placebo effect also plays an important role in NcplP [81]. Some studies have reported that naltrexone may relieve pain [15,27]. Lastly, cannabis-based medicine (CBM) has been assessed in terms of whether it can alleviate NcplP, with reports showing a reduction in pain severity [43,90,116]; however, it carries the risk of adverse effects and addiction [45].

## 6. Primarily Clinical Trials

Although, up-to-date, a wide range of treatments has been investigated and used in clinical practice, and the research on effective treatments for NcplP is still ongoing. Furthermore, due to diagnostic difficulties and complex pathophysiology, only a few clinical trials focused primarily on NcplP have been conducted. Acetyl-L-carnitine has been reported to be potentially beneficial in some diseases associated with NcplP [34]. The role of polyunsaturated fatty acids in pain regulation has also been assessed [42]. Self-reported methods have been shown to be useful when dealing with urologic chronic pelvic pain syndrome [88]. Pain catastrophizing, which is believed to cause NcplP, has been investigated in patients with rheumatoid arthritis. It has been proven that the presence of catastrophizing pain is a barrier when trying to achieve remission in rheumatoid arthritis [95]. Achilles tendinopathy has also been studied, but the presence of NcplP was not confirmed in the examined group [117].

Another helpful tool was developed to assess patients at risk for chronic postsurgical pain. During validation, a painful cold within 2 weeks after surgery was identified as a strong predictor of the development of pain chronicity [118]. There is a report on the development of a screening tool in the field of chronic pain that takes into account NcplP [119]. Currently, there is an ongoing clinical trial investigating a drug-free approach to fibromyalgia [64].

Regarding pathophysiology, the role of toll-like receptor 4 cytokine/chemokine release has been evaluated in the context of an immunological approach [120]. In one paper, the use of the rat grimace scale was reported to be able to predict the presence of NcplP in reserpine-induced fibromyalgia-like rats. The authors state that it can be helpful in translating the effects of therapeutic interventions between animals and humans [121]. Postinjury stimulation of the wounded area was reported to cause pain conversion into NcplP [28]. Another study by McDonough et al. showed that spinal microglia can transmit NcplP [122]. In both studies, it was emphasized that a sex-dependent mechanism may play a pivotal role in NcplP [28,103,122].

The murine model was conducted on mice with reserpine-induced myalgia (RIM6) and acidified saline intramuscular injections (ASI) as a model of NcplP [123]. In addition, the mouse model was used in another study that evaluated the role of sigma-1 receptor (σ1R) ligands with the use of BD1063. The effect of BD1063, which is a σ1R antagonist, was found to provide long-term pain relief in ASI that was even longer than pregabalin in the RIM6 model [124]. SR 57227A, tested in an animal model, was reported as a potential therapeutic agent for chronic pain conditions, e.g., fibromyalgia. Furthermore, it does not have addictive potential [125]. Furthermore, the efficacy of pregabalin, acetaminophen and duloxetine was evaluated in animal models [126]. To our best knowledge, there is unfortunately no other study that has directly investigated the role of palmitoylethanolamide (PEA) in NcplP. PEA was found to be a mechanism of action that includes mast cells and basophils and has been proven to exhibit a neuroprotective effect in cerebellar granule cells [127]. However, taking into account the pathophysiology of NcplP and the cannabimimetic actions of PEA, it is likely to be worth investigating whether PEA will elicit analgesia [127,128,129]. In addition to that, PEA was shown to suppress inflammation [127,129,130]. Finally, the form and route of PEA administration may play an important role [131,132].

## 7. Conclusions

NcplP is the latest pain phenotype to be recognized in clinical practice. It is postulated to be present in many different pathological conditions or diseases belonging to different groups, ranging from fibromyalgia to headaches and even cancers. Despite the rapid development of diagnostic tools, there is no standard approach to the diagnosis of NcplP, so its prevalence can be underestimated. On the other hand, the evidence on the specific characteristics that a patient with NcplP presents and the predominance of pain phenotyping is growing rapidly and helps physicians to select effective therapeutic approaches.

This review has identified several gaps in current knowledge and clinical practice, such as the lack of NcplP-specific diagnostic tools or standard-of-care treatments for NcplP-associated symptoms. Further research is needed to better understand the mechanisms that contribute to the development of NcplP, which can be used to determine treatment standards and improve quality of life in patients with NcplP.

## Data Availability

Data are contained within the article.
